# Hybrid Inorganic–Organic Complexes of Zn, Cd, and Pb with a Cationic Phenanthro-diimine Ligand

**DOI:** 10.1021/acs.inorgchem.2c02867

**Published:** 2022-11-22

**Authors:** Diana Temerova, Tai-Che Chou, Kristina S. Kisel, Toni Eskelinen, Niko Kinnunen, Janne Jänis, Antti J. Karttunen, Pi-Tai Chou, Igor O. Koshevoy

**Affiliations:** † Department of Chemistry, University of Eastern Finland, Joensuu 80101, Finland; ‡ Department of Chemistry, National Taiwan University, Taipei 10617, Taiwan; § Department of Chemistry and Materials Science, Aalto University, Aalto 00076, Finland

## Abstract

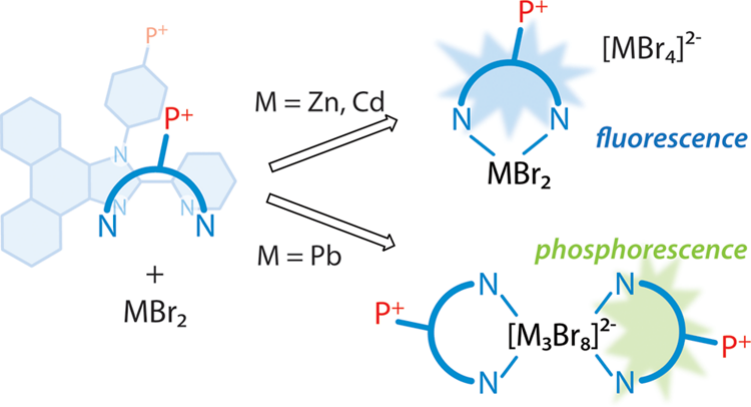

The phosphonium-decorated phenanthro-imidazolyl pyridine ligand, **LP**^**+**^Br, readily reacts with zinc(II) and cadmium(II) bromides to give inorganic–organic zero-dimensional compounds [**LP**^**+**^ZnBr_2_]_2_[ZnBr_4_] (**1**) and [(**LP**^**+**^)_2_Cd_2_Br_4_][CdBr_4_] (**2**), respectively, upon crystallization. These salts are moderately fluorescent in the solid state under ambient conditions (λ_em_ = 458 nm, Φ_em_ = 0.11 for **1**; λ_em_ = 460 nm, Φ_em_ = 0.13 for **2**). Their emission results from spin-allowed electronic transitions localized on the organic component with the negligible effect of [MBr_4_]^2–^ and MBr_2_ units. Contrary to ionic species **1** and **2**, lead(II) bromide affords a neutral and water-stable complex [(**LP**^**+**^)_2_Pb_3_Br_8_] (**3**), showing weak room-temperature phosphorescence arising from spin–orbit coupling due to the heavy atom effect. The emission, which is substantially enhanced for the amorphous sample of **3** (λ_em_ = 575 nm, Φ_em_ = 0.06), is assigned to the intraligand triplet excited state, which is a rare phenomenon among Pb(II) molecular materials.

## Abstract 2

A phosphonium-decorated diimine chromophore forms zero-dimensional hybrid complexes with zinc(II) and cadmium(II) bromides, which demonstrate intraligand fluorescence in the solid state. On the contrary, the use of lead(II) bromide leads to a zwitterionic compound with the [Pb_3_Br_8_]^2−^ cluster core, showing room-temperature intraligand phosphorescence.

## Introduction

Hybrid materials composed of ionic organic and metal halide components, where metal is a post-transition element from groups 12–15, demonstrate remarkably rich photophysical behavior and have been intensely investigated due to their potential optoelectronic applications, covering electroluminescent devices, solar cells, detectors, and sensors.^1−5^ Optical properties of such materials are generally defined by the architecture and composition of halometalate moieties along with characteristics of the lattice, which opens wide possibilities for tuning physical performance once structure–property relationships are understood. In particular, the large variability of organic ionic blocks affects connectivity within an anionic metal halide framework, which can adopt from zero- (0D) to three-dimensional (3D) topology in the crystalline state.^6,7^ Utilization of sterically demanding cations typically results in the formation of 0D hybrids, i.e., those comprising individual metal halide anions of mononuclear and cluster nature, spatially separated by organic “insulators”.^8−11^ Due to relatively nonrigid structural confinement imposed by the lattice, these compounds often exhibit broad photoemission with large Stokes shifts and can reach exceptionally high quantum yields.^10,12−18^

**Scheme 1:**
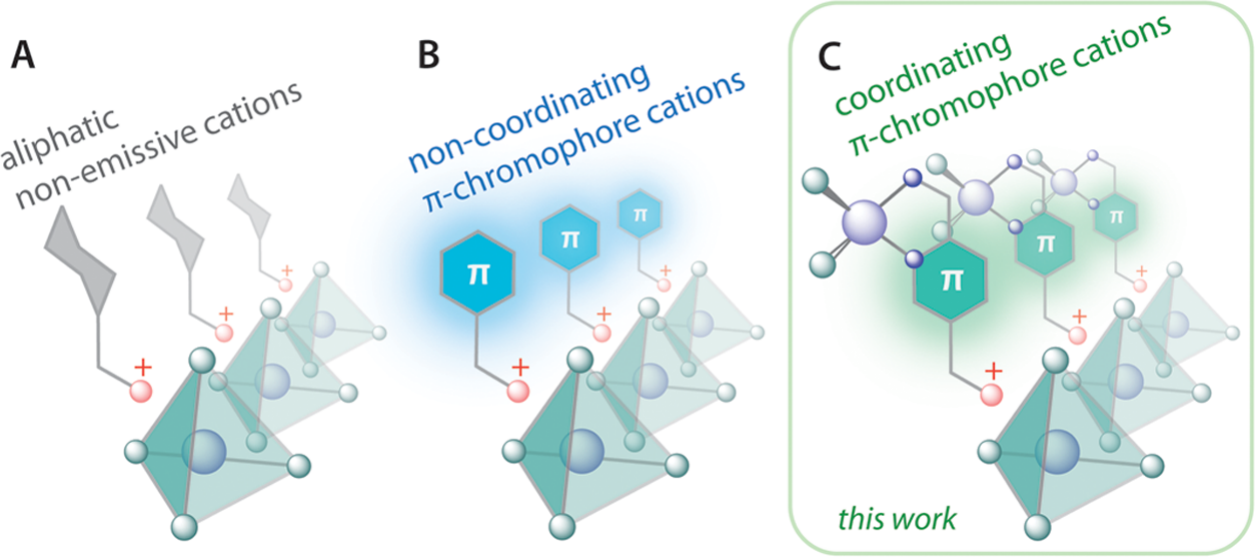
Illustration of Inorganic–Organic Hybrids Based on Different Types of Organic Cations: (A) Aliphatic Nonemissive Cations; (B) Noncoordinating Cations with Chromophore Groups; and (C) Chromophore Cations with Coordinating Function

The majority of hybrid systems include nonconjugated organic cations, which play a negligible electronic role and influence photophysics by governing the crystal packing, the subtle structural features, and the local environment of the metal halide anionic components (Scheme 1A). Nevertheless, recently, there has been an increasingly growing number of reports on organic–inorganic compounds, which employ aromatic chromophores as cations having a noticeable or even dominating contribution to the luminescence of such ionic materials (Scheme 1B). Thus, the resonant energy transfer from one-dimensional (1D) lead chloride chains to organic molecules was proposed for broad-band luminescence demonstrated by [(aminoquinoline)H_2_][PbCl_4_] salt.^19^ The prevailing blue-to-white fluorescence originating from quaternized organic bases (various pyridines; pyrimidines; ethylene-, benzyl-, and stilbenyl amines; etc.) is observed for [MX_3_(dmso)] (M = Zn, X = Br; M = Cd, X = I),^20^ [ZnX_4_]^2–^,^13,21,22^ [InBr_4_]^−^,^23^ [PbCl_5_]^3–^,^24^ and [PbCl_3_]^−^^25^ derivatives, the quantum yields and photostability being substantially higher than those for parent organic halide salts. Alternatively, the related compounds [(aminopyridinium)][HgBr_4_],^13^ [diammoniumdiphenyl sulfone][SnCl_6_],^26^ and [bis(pyridinium)propane][Pb_2_X_6_]^27^ display broad-band luminescence due to the interplay of inorganic (self-trapped) and organic-localized excitons.

Optical properties of the inorganic–organic ionic compounds are further broadened by populating the triplet excited states, leading to room-temperature phosphorescence.^3,28^ The lifetimes and quantum yields of organic ultralong photoemission can be readily modulated by means of the external heavy atom effect, i.e., varying the nature of the constituting halometalates, that was shown for complexes [benzoquinolinium][Pb_2_X_5_],^29^ [PPh_4_][ZnX_4_],^30^ and [PPh_4_][Cd_2_X_6_],^31^ the latter for X = Br reaches notably high Φ_em_ = 63% (τ = 37.85 ms).

Another approach to diversify the structural organization and photophysical characteristics of hybrid compounds potentially relies on the employment of cationic chromophores with coordinating function and yet remains virtually unexplored (Scheme 1C). The aromatic ligands, e.g., strongly binding N-donors (bi-/tripyridine, phenanthroline, and other heterocycles), decorated with remote positively charged groups (pyridinium/quinolinium, phosphonium, ammonium, etc.) are not exceptional and have been used in the design of a number of phosphorescent complexes of Ru, Re, Ir, and Pt for bioimaging applications,^32^ and some Cu(I) zwitterionic iodide clusters with delayed fluorescence.^33,34^ These sorts of constructing elements, however, have not penetrated the field of inorganic–organic systems built of post-transition metals, except sporadic examples like [(quinolinium terpyridine)ZnBr_2_][ZnBr_4_(dmso)],^35^ confirming the preparative feasibility of the given strategy.

From the photophysical viewpoint, it is well known that group 12 metal halides, which are used as starting reagents for the fabrication of halometalate-based hybrids, and the related salts MX_2_ (M = Zn, Cd) readily form fluorescent complexes with chelating N- and O-donors (e.g., N-heterocyclic and Schiff bases, salen-type ligands, etc.) adopting 4-, 5-, and 6-coordination numbers.^36−45^ Coordination of the metal centers may lead to the conformational modification^46^ and substantial perturbation of the electronic structure of organic molecules, resulting in, e.g., enhanced intramolecular charge transfer,^39,40,47^ appearance of stimuli-responsive and sensing ability,^40,43−45,48,49^ suppression of photoinduced electron transfer, and dramatic improvement of quantum efficiency typically accompanied by the bathochromic shift of the emission.^36,39,46,47,50^ On the other hand, the coordinated MX_2_ units tend to participate in intermolecular noncovalent interactions, which have a strong impact on the solid-state arrangement and the corresponding optical properties.^41,42,51^

In comparison to zinc and cadmium, the coordination chemistry of main group metals is less developed for the soft N-heterocyclic ligands and is illustrated by pseudo-octahedral complexes of Pb(II), forming 1D polymeric chains [Pb(diimine)(μ-X)_2_]*_n_*,^52−56^ the emission energy of which is regulated by aromatic system.^56^

To probe the viability of the concept illustrated in Scheme 1C and to utilize the cationic chromophore ligand in the synthesis of low-dimensional metal hybrids, as a case study, we have chosen easy-to-obtain and -modify phenanthrene fused with the chelating imidazolyl-pyridine motif, which readily coordinates to late transition metals as was earlier shown by us and other groups.^42,57,58^ The decoration of this fluorescent diimine ligand with the chemically robust tetraarylphosphonium group affords a bulky metal-binding cation, which was explored in the preparation of hybrid compounds derived from Zn, Cd, and Pb halide units.

## Experimental Section

### General Comments

1-(4-Bromophenyl)-2-(pyridin-2-yl)-1*H*-phenanthro[9,10-*d*]imidazole (**LBr**) was prepared according to the reported procedure.^58^ Other reagents were used as received. Toluene was distilled over Na-benzophenone ketyl under a nitrogen atmosphere prior to use. The solution ^1^H and ^31^P{^1^H} spectra were recorded on a JEOL 500 spectrometer. Microanalyses were carried out in the analytical laboratory of the University of Eastern Finland.

### Triphenyl(4-(2-(pyridin-2-yl)-1*H*-phenanthro[9,10-*d*]imidazol-1-yl)phenyl) Phosphonium Bromide (**LP**^+^Br)

The synthesis was carried out in a pressure tube following the general protocol for the preparation of tetraarylphosphonium salts.^59^ The tube was charged with solid **LBr** (0.5 g, 1.11 mmol), triphenylphosphine (0.291 g, 1.11 mmol), and Pd_2_(dba)_3_ (6.4 mg, 0.007 mmol), which were degassed, and dry toluene (0.7 mL) was added under a nitrogen atmosphere. The reaction mixture was heated to 140 °C for 12 h, resulting in the gradual formation of a precipitate of the phosphonium salt. The suspension was cooled down to room temperature, the solvent was removed by filtration, and the crude product was washed with diethyl ether (2 mL × 5 mL). The solid was dried and further purified by column chromatography (silica gel 70–230 mesh, ⌀4 cm × 15 cm, eluent dichloromethane/methanol 40:1 → 10:1 v/v mixture) to afford a pale creamy amorphous material after evaporation of the volatiles (688 mg, 87%). ^1^H NMR (acetonitrile-*d*_3_, 298 K; δ): 8.86 (d, *J*_HH_ 8.0 Hz, 1H), 8.79 (d, *J*_HH_ 8.0 Hz, 1H), 8.75 (dd, *J*_HH_ 7.8, 1.2 Hz, 1H), 8.39 (dt, *J*_HH_ 7.9, 1.0 Hz, 1H), 8.24 (ddd, *J*_HH_ 4.8, 1.8, 0.9 Hz, 1H), 7.97–7.92 (m, 5H), 7.92–7.86 (m, 3H), 7.82–7.74 (m, 13H), 7.70 (ddd, *J*_HH_ 8.4, 7.1, 1.5 Hz, 1H), 7.60 (ddd, *J*_HH_ 8.4, 7.0, 1.3 Hz, 1H), 7.39 (ddd, *J*_HH_ 8.3, 7.0, 1.2 Hz, 1H), 7.31 (ddd, *J*_HH_ 7.6, 4.8, 1.2 Hz, 1H), 7.22 (dd, *J*_HH_ 8.4, 1.0 Hz, 1H). ^31^P{^1^H} NMR (acetonitrile-*d*_3_, 298 K; δ): 23.7 (s). ^1^H NMR (*N*,*N*-dimethylformamide-*d*_7_, 298 K; δ): 9.01 (d, *J*_HH_ 8.2 Hz, 1H), 8.95 (d, *J*_HH_ 8.4 Hz, 1H), 8.80 (dd, *J*_HH_ 8.0, 1.3 Hz, 1H), 8.48 (dt, *J*_HH_ 7.9, 1.0 Hz, 1H), 8.34 (ddd, *J*_HH_ 4.8, 1.7, 0.9 Hz, 1H), 8.29–8.25 (m, 2H), 8.20–8.14 (m, 2H), 8.10–7.94 (m, 15H), 7.88–7.81 (m, 2H), 7.75 (ddd, *J*_HH_ 8.4, 7.0, 1.5 Hz, 1H), 7.64 (ddd, *J*_HH_ 8.3, 7.0, 1.2 Hz, 1H), 7.45–7.38 (m, 2H), 7.28 (dd, *J*_HH_ 8.3, 1.0 Hz, 1H). ESI-MS (*m*/*z*): [M]^+^ 623.2267 (calcd 623.2256). Anal calcd for C_44_H_31_BrN_3_P: C, 74.16; H, 4.38; N, 5.90. Found: C, 73.84; H, 4.69; N, 5.82.

### [LP^+^ZnBr_2_]_2_[ZnBr_4_] (**1**)

A solution of ZnBr_2_ (47 mg, 0.21 mmol) in ethyl acetate (5 mL) was added to a solution of **LP**^**+**^Br (100 mg, 0.14 mmol) in acetonitrile (20 mL). The reaction mixture was stirred for 30 min, and a nearly transparent solution was filtered and left at room temperature for slow evaporation to give a white crystalline material (124 mg, 84%). ^1^H NMR (acetonitrile-*d*_3_, 1.69 × 10^–3^ M, 298 K; δ): δ 8.93 (d br, *J*_HH_ 7.8 Hz, 1H), 8.90 (d, *J*_HH_ 8.3 Hz, 1H), 8.85 (d, *J*_HH_ 8.3 Hz, 1H), 8.70 (s br, 1H), 8.19–8.05 (m, 4H), 8.00–7.93 (m, 4H), 7.88–7.77 (m, 15H), 7.70–7.64 (m, 2H), 7.43 (ddd, *J*_HH_ 8.2, 7.1, 1.0 Hz, 1H), 7.05 (d, *J*_HH_ 7.4 Hz, 1H). ^31^P{^1^H} NMR (acetonitrile-*d*, 298 K; δ): 23.8 (s). Anal calcd for C_88_H_62_Br_8_N_6_P_2_Zn_3_·CH_3_CN: C, 50.47; H, 3.06; N, 4.58. Found: C, 50.63; H, 3.11; N, 4.76.

### [(LP^+^)_2_Cd_2_Br_4_][CdBr_4_] (**2**)

CdBr_2_ (42 mg, 0.15 mmol) and **LP**^**+**^Br (71 mg, 0.10 mmol) were suspended in a mixture of acetonitrile (12 mL) and methanol (6 mL). The suspension was heated at ca 60 °C under stirring until complete dissolution of the solids (ca 15 min). The resulting solution was filtered and concentrated by slow evaporation at room temperature to give a white crystalline material (**2**), which was washed with acetonitrile (5 mL) and diethyl ether (2 mL × 5 mL) and dried (94 mg, 85%). ^1^H NMR (acetonitrile-*d*_3_, 298 K; δ): 8.89 (d, *J*_HH_ 6.8 Hz, 1H), 8.85 (d, *J*_HH_ 8.2 Hz, 1H), 8.79 (dd, *J*_HH_ 8.1, 1.3 Hz, 1H), 8.69 (s br, 1H), 7.99–7.92 (m, 7H), 7.89 (td, *J*_HH_ 7.8, 1.7 Hz, 1H), 7.83–7.77 (m, 13H), 7.75–7.69 (m, 2H), 7.63 (ddd, *J*_HH_ 8.3, 7.1, 1.2 Hz, 1H), 7.53 (dd, *J*_HH_ 8.7, 3.6 Hz, 1H), 7.38 (ddd, *J*_HH_ 8.2, 7.1, 1.1 Hz, 1H), 7.09 (d, *J*_HH_ 8.4 Hz, 1H). ^31^P{^1^H} NMR (acetonitrile-*d*_3_, 298 K; δ): 23.8 (s). Anal calcd for C_88_H_62_Br_8_Cd_3_N_6_P_2_: C, 47.15; H, 2.79; N, 3.75. Found: C, 47.08; H, 2.85; N, 3.91.

### [(LP^+^)_2_Pb_3_Br_8_] (**3**)

A solution of **LP**^**+**^Br (70 mg, 0.10 mmol) in acetonitrile (1.5 mL) was added to a solution of PbBr_2_ (51 mg, 0.14 mmol) in dimethylformamide (3 mL). The resulting mixture was gently refluxed for ca 10 min, giving a nearly transparent pale yellow solution, which was filtered. Gas-phase diffusion of water into this solution at room temperature for 1 week produced **3** as a pale yellow crystalline material, which was washed with acetonitrile (2 mL × 5 mL) and diethyl ether (2 mL × 5 mL) and dried (85 mg, 71%). Anal calcd for C_88_H_62_Br_8_N_6_P_2_Pb_3_: C, 41.84; H, 2.47; N, 3.33. Found: C, 42.04; H, 2.55; N, 3.75.

### X-ray Structure Determinations and Powder Measurements

The crystals of **1**–**3** were immersed in cryo-oil, mounted in a Nylon loop, and measured at a temperature of 150 K. The X-ray diffraction data were collected with a Bruker Kappa Apex II diffractometer using Mo Kα (λ = 0.71073 Å) radiation. The APEX2^60^ program package was used for cell refinements and data reductions. A numerical or semiempirical absorption correction (SADABS)^61^ was applied to all data. The structures were solved by direct methods using the SHELXS-2018^62^ program with the WinGX^63^ graphical user interface. Structural refinements were carried out using SHELXL-2018.^62^

Some of the crystallization solvent molecules in **2** could not be resolved unambiguously. The acetonitrile solvent molecules were refined with an occupancy of 0.5. The contribution of the missing solvent to the calculated structure factors was taken into account by a SQUEEZE routine^64^ of PLATON.^65^ The missing solvent was not taken into account in the unit cell content. All non-H atoms were anisotropically refined, and all hydrogen atoms were positioned geometrically and constrained to ride on their respective parent atoms with C–H = 0.95–0.98 Å and *U*_iso_ = 1.2–1.5*U*_equiv_ (parent atom). The crystallographic details are summarized in Table S1 (Supporting Information).

Powder X-ray powder diffraction (XRD) patterns were recorded with a Bruker Advance D8 diffractometer using a Cu Kα (1.54184 Å) radiation source (Bruker: 40 kV/ 40 mA). Divergence and receiving slits of 1.0 mm were used together with a Ni filter in the measurements. Prior to measurement, a sample was placed on a Si single-crystal zero background sample holder. The diffraction patterns were scanned from 5 to 90° in a 2θ scale using the locked couple technique in Bragg–Brentano geometry. A collection time of 2 s was used together with a step size of 0.05° per step.

### Photophysical Measurements

The excitation and emission spectra for the solid samples were recorded with an Edinburgh FLS980 spectrofluorometer. Photoluminescence quantum yields and the absorption spectra of solid materials were measured by an integrating sphere (F-M01, Edinburgh) set in the sample chamber of the spectrometer (Edinburgh FLS980). Emission lifetimes within 1 ns–20 μs were collected by an inbuilt, time-correlated single photon counting (TCSPC) system (Edinburgh FLS980) coupled with a synchronized diode laser (EPL-375) as the pumping source. For phosphorescence lifetimes longer than 50 μs, an intensified charge-coupled detector (PI-MAX ICCD) coupled with a Q-switch laser was used instead. Both timings of ICCD and tunable laser are triggered by a pulse generator (DG-535, Stanford Research System) with signal jittering less than 5 ns. The excitation pulse was generated by an LT-2134 (532 nm, SHG of Nd:Yag laser, LOTIS Tii) followed by an LT-2211 tunable laser (345–532 nm, LOTIS Tii), and 365 nm was chosen as the excitation source with an fwhm of ∼20 ns.

### Computational Details

Quantum chemical studies on all complexes were carried out using density functional theory (DFT). The PBE0 hybrid density functional^66,67^ in combination with the def2-TZVP basis set^68^ and the corresponding scalar-relativistic effective core potential^69^ were applied for Cd and Pb atoms. The full model of Cd complex **2** was optimized using a QM/MM approach within the ONIOM framework,^70^ since attempts to locate a minimum geometry with the isolated single-molecule model were unsuccessful. In the QM/MM model, the crystal structure was expanded in three dimensions and the central molecule was assigned as the QM part, while the surroundings were kept frozen and treated with MM using the UFF force field.^71^ Electronic embedding with the QEq scheme^72^ was used to account for polarization of the QM region. The QM/MM optimization was performed using Gaussian 16.^73^ All other calculations were performed with Orca 5.0.3^74^ using single-molecule models in the optimizations. The geometry of the first excited singlet state was optimized with TD-DFT, while the ground state and first excited triplet state were optimized with DFT. For compound **3**, the implicit CPCM solvation model^75^ with toluene as the solvent was used because the geometry optimization did not converge in a vacuum environment. To confirm that the obtained geometries corresponded to a true local minimum on the potential energy surface, frequency calculations were performed for all optimized structures. Furthermore, to study the excitation and emission behavior, single-point TD-DFT calculations were performed for all optimized structures using the range-separated LRC-ωPBEh^76^ functional together with the def2-TZVP basis set. The resolution of identity approximation together with the chain of spheres for exchange approximation (RIJCOSX)^77,78^ was used in all calculations with Orca to speed up the calculations.

## Results and Discussion

### Synthesis and Characterization

**Scheme 2:**
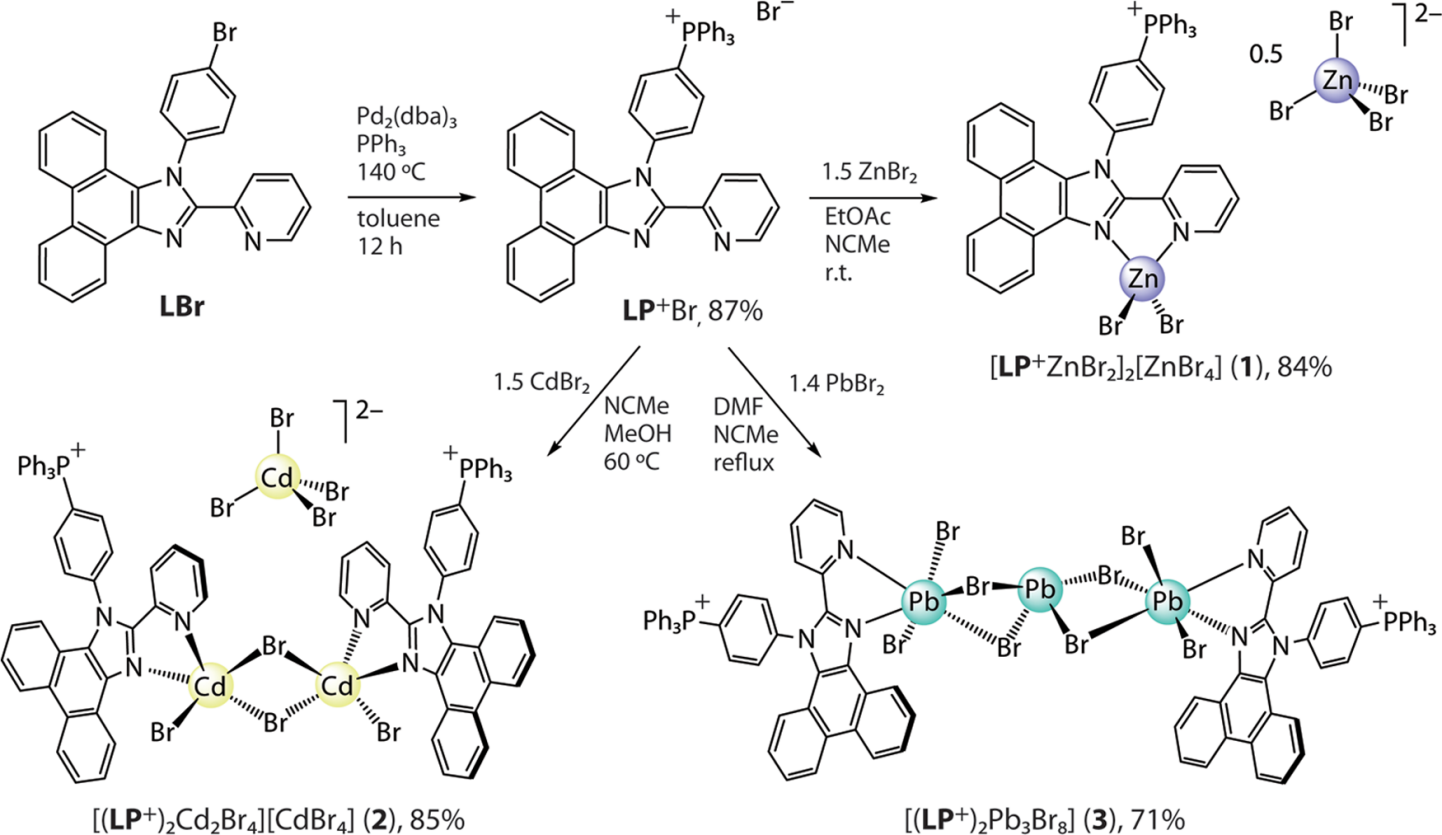
Synthesis of the Cationic Diimine Ligand and Its Zn, Cd, and Pb Complexes

The tetraarylphosphonium unit was chosen due to its stability, bulkiness, and accessible synthesis, the conditions of which tolerate the N-heterocycles. Functionalization of the phenanthro-imidazolyl pyridine was performed according to the general method by Marcoux (Scheme 2 and the Supporting Information).^59^ The palladium-catalyzed reaction of the bromoaryl precursor (**LBr**)^58^ with a stoichiometric amount of triphenylphosphine afforded the corresponding phosphonium bromide (**LP**^**+**^Br) in good yield. Hybrid complexes of zinc(II) and cadmium(II) were obtained by treating the ZnBr_2_ and CdBr_2_ salts with **LP**^**+**^Br in ethyl acetate/acetonitrile and methanol/acetonitrile mixtures, respectively. Slow evaporation of the resulting solutions at room temperature produced colorless crystals of ionic species [**LP**^**+**^ZnBr_2_]_2_[ZnBr_4_] (**1**) and [(**LP**^**+**^)_2_Cd_2_Br_4_][CdBr_4_] (**2**) suitable for X-ray diffraction analysis. Due to the lower solubility of PbBr_2_, it was reacted with **LP**^**+**^Br in a dimethylformamide/acetonitrile solvent system. Subsequent gas-phase diffusion of water into the reaction mixture gave a neutral complex [(**LP**^**+**^)_2_Pb_3_Br_8_] (**3**) as a pale yellow crystalline material (Scheme 2). The formation of **1** and **3** was found to be selective and rather independent of the ratio of starting reagents, while in the case of **2**, proper stoichiometry (**LP**^**+**^Br/CdBr_2_ 2:3) has to be obeyed to minimize crystallization of side products. A slight excess of **LP**^**+**^Br in the synthesis of **3** was used to avoid precipitation of PbBr_2_. The iodide analogues of **1** and **2** can also be obtained in a similar fashion, but the crystalline materials visibly degrade under exposure to light and are virtually nonluminescent under ambient conditions and thus were omitted from this work. In yet another approach, we were unable to prepare the lead iodide complex in a reproducible manner and in pure form.

**Figure 1:**
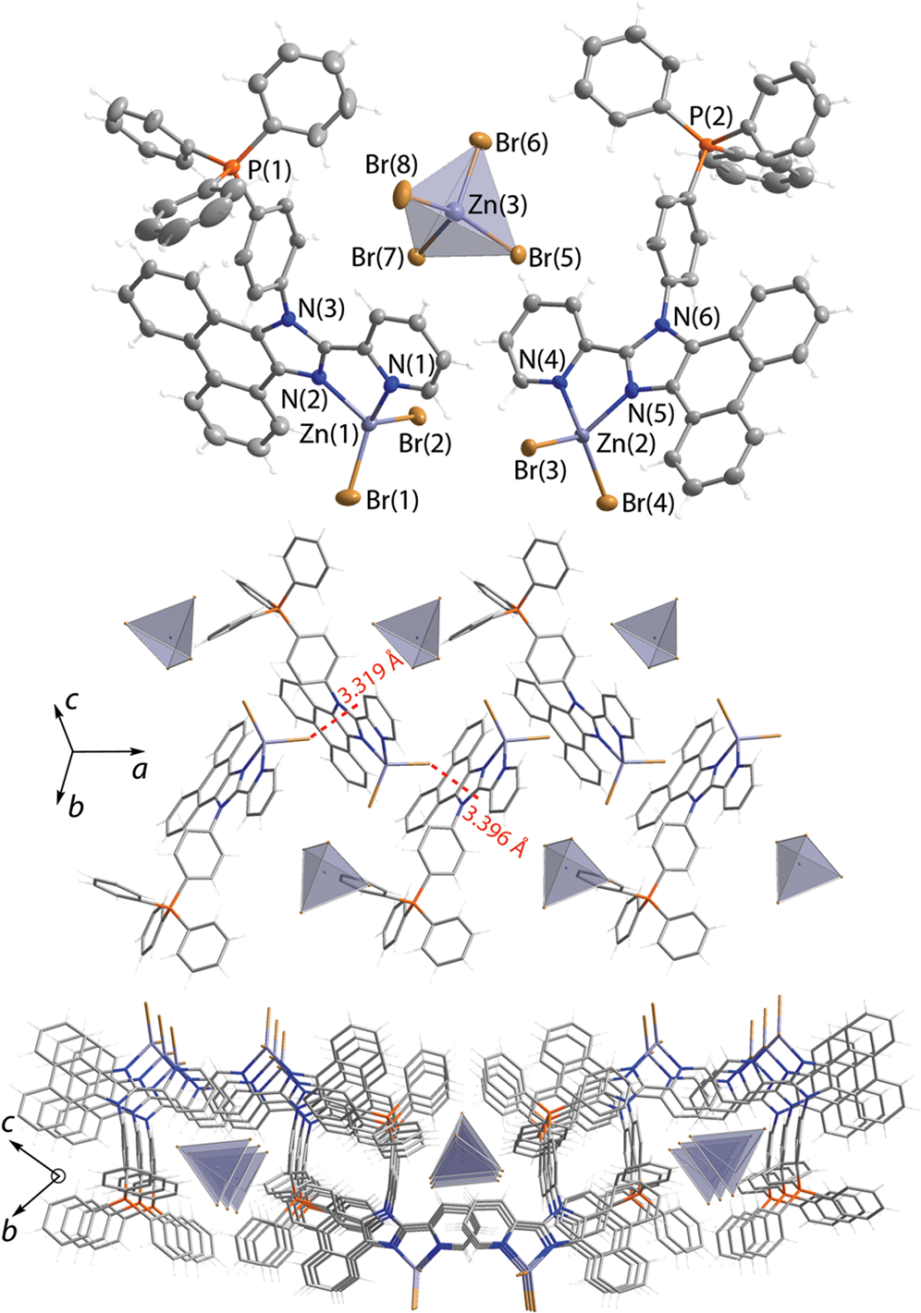
Molecular (top) and packing extended (middle and bottom) structures of salt [**LP**^**+**^ZnBr_2_]_2_[ZnBr_4_] (**1**); thermal ellipsoids are shown at 50% probability.

**Figure 2:**
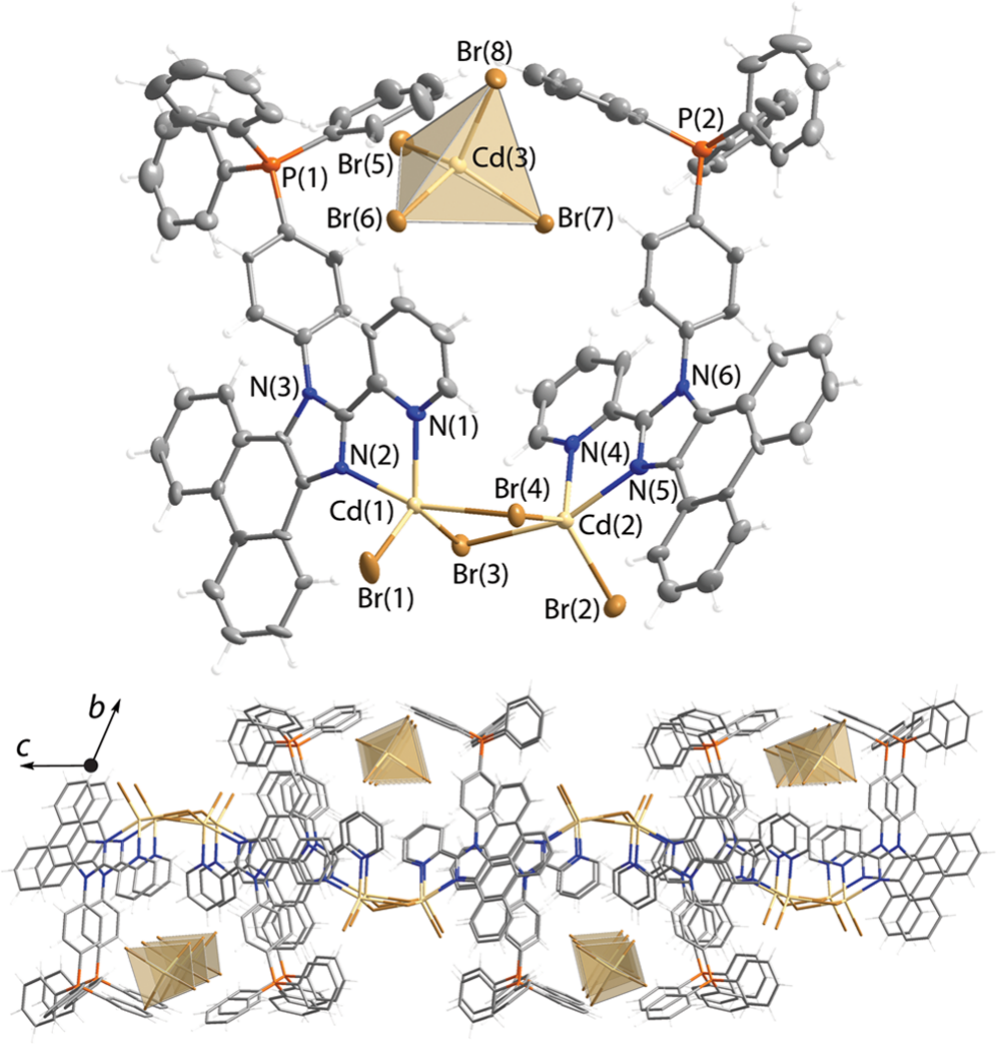
Molecular (top) and packing extended (bottom) structures of salt [(**LP**^**+**^)_2_Cd_2_Br_4_][CdBr_4_] (**2**); thermal ellipsoids are shown at 50% probability.

**Figure 3:**
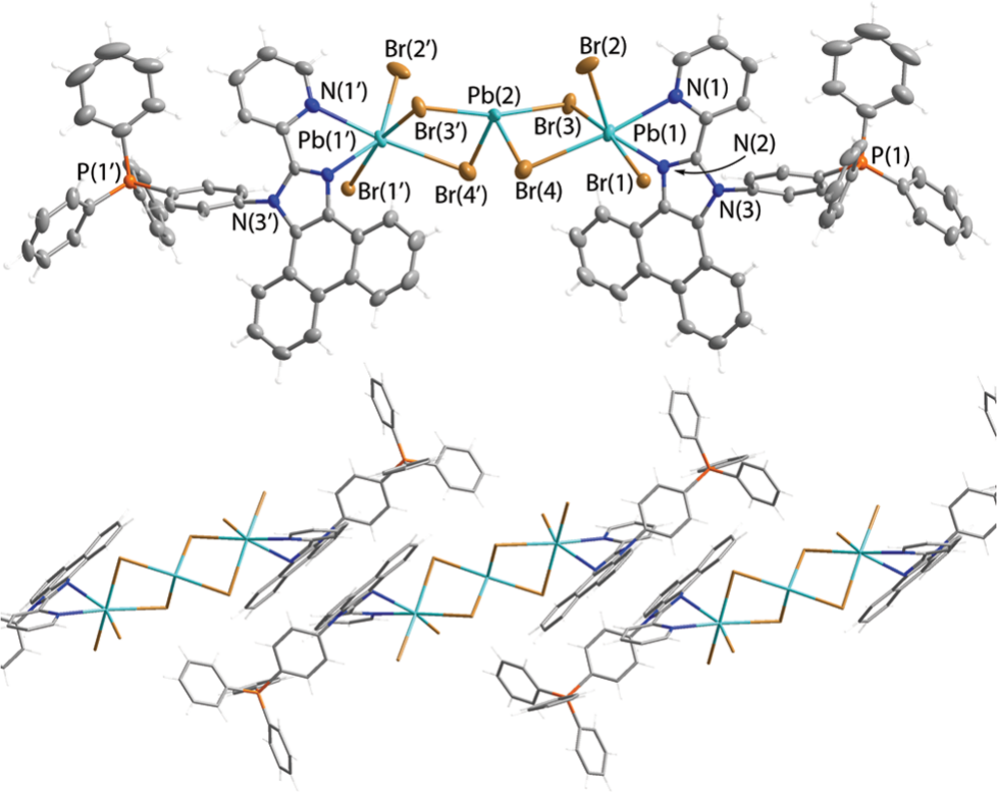
Molecular (top) and packing extended (bottom) structures of complex [(**LP**^**+**^)_2_Pb_3_Br_8_] (**3**); thermal ellipsoids are shown at 50% probability.

The structures of **1**–**3** determined by XRD analysis are depicted in Figures 1–3, and selected bond lengths and angles are listed in Table S2 (SI). The repeating motif of salt **1** is composed of individual inorganic dianions [ZnBr_4_]^2–^ and two crystallographically nonequivalent metal–organic cations [**LP**^**+**^ZnBr_2_]^+^. In turn, the latter constituents comprise the phosphonium-decorated ligand, coordinated to the zinc dibromide unit via chelating imidazolyl-pyridine function. In this cationic complex, the metal center expectedly adopts pseudo-tetrahedral coordination geometry, analogously to the neutral predecessor compounds we described earlier^42^ and to other related [Zn(diimine)Hal_2_] species.^37,41,46,79^

Cadmium complex **2** reveals the same stoichiometry as that found in **1**, [**LP**^**+**^MBr_2_]_2_[MBr_4_], and is topologically similar to the zinc congener (Figure 2). The slightly distorted [CdBr_4_]^2–^ tetrahedron is placed in voids formed by tetraarylphosphonium groups, which is apparently driven by the electrostatic attraction. The [**LP**^**+**^CdBr_2_]^+^ cations dimerize via the nonsymmetrical bromide bridges (μ_2_-Br–Cd distances are 2.640(2), 2614(2), 2.861(2), and 2.802(2) Å; Table S2) that provides pentacoordinate environment of Cd(II) ions and evidently follows a preference of [Cd(diimine)Hal_2_] units to have a higher coordination number than that of Zn(II) analogues.^80−82^

The [MBr_4_]^2–^ anions in **1** and **2** form networks of hydrogen bonds with organic molecules and are well separated from each other (Figures 1 and 2); the distances between the bromine atoms of adjacent anions exceed 10 Å (**1**) and 6.6 Å (**2**), which are likely too long for appreciable electronic coupling between these inorganic components.

In contrast to ionic compounds **1** and **2**, lead(II) bromide produced overall neutral molecular entity **3**, which can be seen as a zwitterionic complex built of two cationic ligands coordinated to an anionic bromoplumbate cluster featuring a discrete [Pb_3_Br_8_]^2–^ core (Figure 3); the complex conceptually resembles the [Cu_4_I_6_]^2+^ clusters stabilized by [RP(pyridine)_3_]^+^ ligands.^33^ The anions of the same composition but of somewhat different geometry were found as 1D polymers in salts [PR_4_]_2_[Pb_3_Br_8_].^83,84^ The trimetallic motif contains a central seesaw [PbBr_4_]^2–^ fragment, which is linked to the lateral PbBr_2_ units via bromide bridges. The geometry of tetracoordinate [PbBr_4_]^2–^ is known for *n*s^2^ metal halides^2,9^ and is derived from a trigonal bipyramid with a 6s^2^ electron pair of Pb(II) being in the equatorial plane.^85,86^ The bond distances from the bridging halides Br(3) and Br(4) to the pendant Pb(1) atom (Br(3/4)–Pb(1) = 3.0893(4) and 3.2068(8) Å) are visibly longer than those within the [PbBr_4_]^2–^ block (Br(3/4)–Pb(2) = 2.9932(4) and 2.7250(5) Å). Such deviation of μ_2_-Br–Pb separations due to nonsymmetrical coordination has been described for other bromoplumbate clusters.^87,88^

The binding of the **LP**^**+**^ ligand to each of the PbBr_2_, together with μ_2_-bromides, completes the highly distorted octahedral environment of the Pb(1) center. This metal atom lies almost in the plane of the pyridine; the Pb(1)–N_py_(1) bond length (2.642(3) Å) indicates relatively strong interaction and is consistent with reported data on the Pb(II) diimine halide complexes.^52,54^ Bonding of the Pb(1) atom to the diimine function is accompanied by a substantial twisting of the pyridine ring with respect to the imidazole; the corresponding dihedral angle between the planes of these heterocycles equals 33.8° (Figure 3). This might be caused, to a large extent, by intermolecular arrangement, which is probably defined by multiple Br^···^H contacts (2.73–3.10 Å) due to the Coulombic forces between the bromide ligands and the positively charged tetraarylphosphonium motifs. As a result, the Pb(1) atom is substantially displaced from the plane of imidazole, leading to a longer Pb(1)–N_imi_(2) distance (2.759(3) Å). This effect can be attributed to the proximity of the lone pair of Pb(II), which causes the elongation and weakening of the Pb–N_imi_ bonding interaction.^89^

The ^1^H NMR spectra recorded for compounds **1** and **2** in acetonitrile-*d*_3_ at room temperature confirm the presence of complex cations [**LP**^**+**^MBr_2_]^+^ in solution (Figure S1), although broadening of the signals for salt **1** upon dilution suggests some dynamic process. This is likely caused by easy dissociation of MBr_2_ units in coordinating solvent and is supported by electrospray ionization (ESI)-mass spectrometry (MS) measurements, which show only the signal of the free ligand. Complex **3** is scarcely soluble in most organic solvents. Its proton spectrum in a dimethylformamide-*d*_6_ solution is identical to that of the free ligand, indicating complete disintegration of the aggregate. Nevertheless, the dissociation does not lead to degradation of the mixture and **3** is efficiently reassembled upon crystallization or slow precipitation with water.

### Photophysical Studies and Theoretical Analysis

For the free ligand in a dichloromethane solution, the lowest energy absorption bands (330–360 nm, Figure S2) can be assigned to the predicted S_0_ → S_1_ transition (λ = 340 nm) from density functional theory (DFT), which mainly involves charge transfer (ILCT) from imidazole to phosphonium-bearing phenylene and the empty orbital of the P–C bond (Figure S3). The radiative relaxation to the ground state S_1_ → S_0_ (λ = 424 nm, Table S3) has virtually identical CT character. In accordance with theoretical results, the observed ILCT fluorescence of **LP**^**+**^Br (λ_em_ = 422 nm, Φ_em_ = 0.1, Figure S2) lacks vibronic progression and demonstrates substantial Stokes shift. This behavior markedly differs from that of the neutral analogue **L** (2-pyridyl-1*H*-phenanthro[9,10-*d*]imidazole) with largely phenanthrene-centered structured emission in the UV region (λ_em_ = 370, 388 nm, Φ_em_ = 0.37).^58^

**Figure 4:**
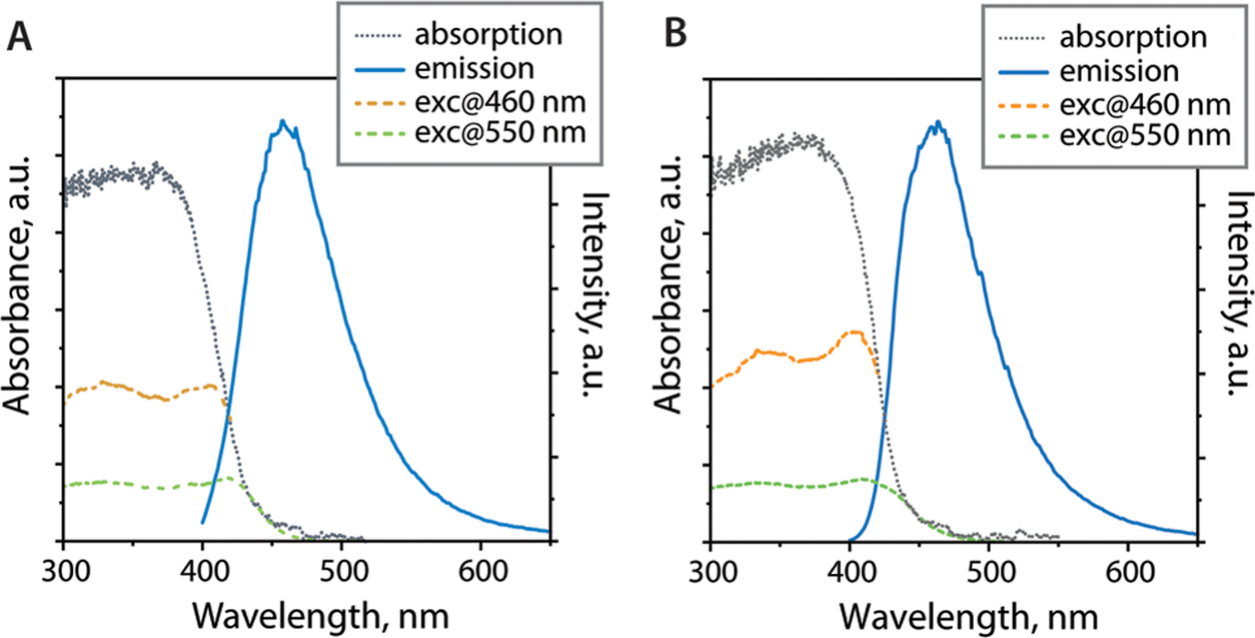
Room-temperature absorption, excitation, and emission (λ_exc_ = 380 nm) spectra of **1** (A) and **2** (B) in the solid state.

Dried crystals of acetonitrile-solvated **1** and **2** show similar room-temperature luminescence (Figure 4 and Table 1). Both compounds are moderate blue emitters, which exhibit broad and structureless signals maximized at 458 (**1**) and 460 nm (**2**) with quantum yields of 0.11 and 0.13, respectively. The luminescence of the ligand salt **LP**^**+**^Br in the solid (λ_em_ = 456 nm, Φ_em_ = 0.04, Figure S2) is substantially weaker, although the peak wavelength is practically unaffected by the presence of the metal units. The average lifetimes (τ_av_ = 1.3 and 1.4 ns) and radiative rate constants (*k*_r_ ≈ ca 10^8^ s^–1^) point to the singlet origin in the excited state, i.e., fluorescence. A similar enhancement of organic luminescence in the hybrid compounds due to a more rigid environment has been reported for other congener materials.^21,22,24^

**Table 1:** Photophysical Properties of Compounds **1**–**3** in the Solid State at 298 K

	λ_fluo_, nm	τ_fluo_, ns	λ_phos_, nm	τ_phos_, μs	Φ_em_	*k*_r,_^b^ s^–1^	*k*_nr,_^b^ s^–1^
**LP**^**+**^Br	456	<1			0.04	>4 × 10^7^^c^	>9 × 10^8^^c^
**1**	458	1.3^a^			0.11	8.5 × 10^7^	6.8 × 10^8^
**2**	460	1.4^a^			0.13	9.3 × 10^7^	6.2 × 10^8^
**3**_cryst_	440	2.1	517, 550	11	0.009	∼8 × 10^2^^d^	∼9 × 10^4 *k*^^d^
**3**_amorph_	460	4.0	575	120	0.06	∼5 × 10^2^^d^	∼8 × 10^3^^d^

a Amplitude-weighted average emission lifetimes for the biexponential decays determined by the equation τ_av_ = ∑*A*_*i*_τ_*i*_, *A*_*i*_ = weight of the *i*th exponent.

b *k*_r_ and *k*_nr_ were estimated by Φ_em_/τ and (1 – Φ_em_)/τ, respectively.

c Estimated approximately due to the inability to accurately determine the lifetime.

d Calculated by neglecting the contribution of fluorescence to the value of Φ_em_.

**Figure 5:**
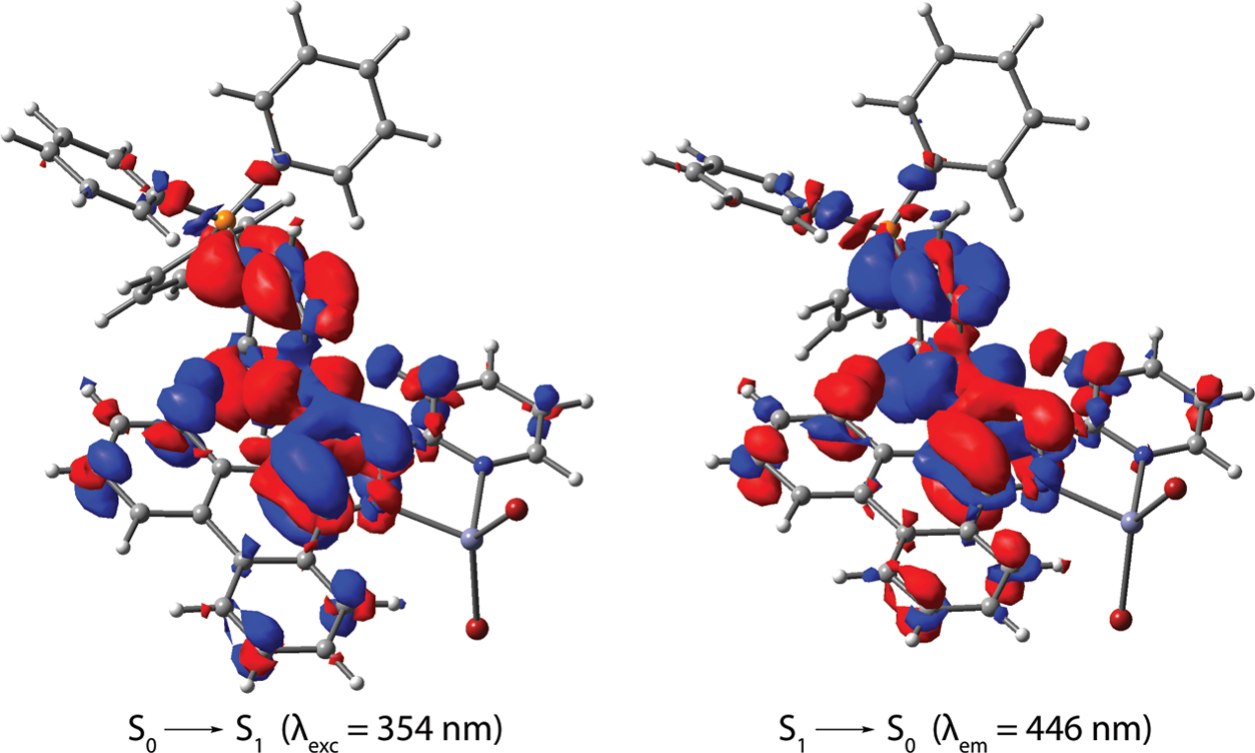
Electron density difference plots for the lowest energy excitation S_0_ → S_1_ and emission S_1_ → S_0_ in the cation [**LP**^**+**^ZnBr_2_]^+^ of compound **1** (isovalue 0.002 a.u., DFT-LRC-ωPBEh method, optimized S_0_ and S_1_ geometries). During the electronic transition, the electron density increases in the red areas and decreases in the blue areas.

Albeit relatively short intermolecular Br^···^π contacts (ca 3.3–3.4 Å, Figure 1) seen in salt **1**, the phosphorescence induced by the external heavy atom effect, which was previously detected for **L**ZnI_2_,^42^ cannot be identified in the room-temperature spectra of **1**. The spectroscopic profiles for **1** and **2** resemble those of the complexes **L**ZnX_2_ (X = Cl, I, OAc).^42^ Thus, the excitation and emission bands of **1** and **2** are likely associated with the [**LP**^**+**^MBr_2_]^+^ cations and are dominated by the organic component, whereas the [MBr_4_]^2–^ anions do not contribute to the S_1_ state. DFT studies of the [**LP**^**+**^MBr_2_]^+^ components (the half model was used for the Cd derivative, Table S3) confirm that the S_0_ ↔ S_1_ electronic transitions have the same nature as those for the free ligand (Figures 5 and S4) and illustrate the insignificant influence of coordinated MBr_2_ units on the optical behavior of **1** and **2**. Compared to the neutral species **L**ZnX_2_, for which the π(phenanthrene) → π*(pyridine) charge transfer was computationally predicted for the low-energy excitation, the appearance of the electron-deficient phosphonium motif decreases the contribution from phenanthrene and pyridine orbitals, substantially changing the character of excitation and emission to π(imidazole) ↔ π*(phenylene-P) transitions.

**Figure 6:**
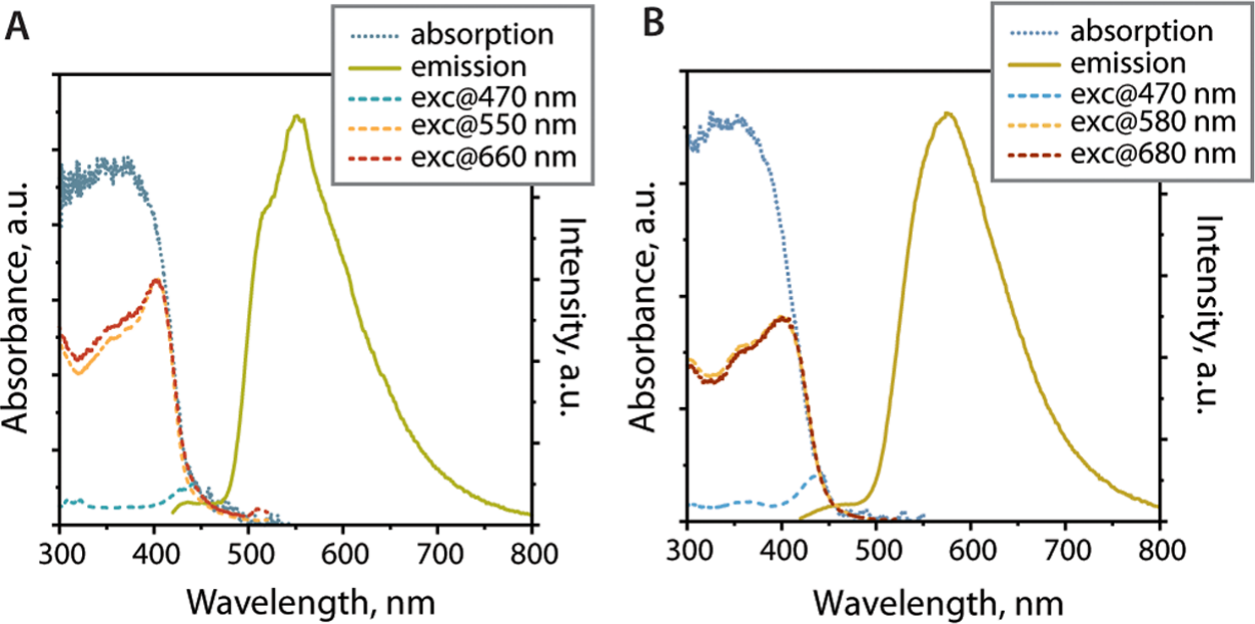
Room-temperature absorption, excitation, and emission (λ_exc_ = 400 nm) spectra of the single crystals (A) and amorphous sample (B) of complex **3**.

Single crystals of complex **3** are poorly luminescent at room temperature under UV excitation, showing the main low-energy band maximized at 550 nm and a very weak high-energy signal around 440 nm (Figure 6) with a total quantum yield of 9 × 10^–3^ (Table 1). The latter minor band has a lifetime of 2.1 ns and can be correlated with the intraligand fluorescence of **1** and **2**. The microsecond lifetime of the prevailing band (τ_obs_ = 11 μs) and the corresponding rate constant (*k*_r_ ≈ 8 × 10^2^ s^–1^) indicate a spin-forbidden process, i.e., phosphorescence. The barely resolved but notwithstanding discernible vibrational structure of the main band points to the triplet state responsible for the emission is localized within the conjugated system of the diimine ligand. This tentative assignment is also implicitly supported by the similarity and broadness of excitation spectra for **1**, **2**, and **3**; for self-trapped exciton emission of zero-dimensional halides, relatively narrow excitation spectra have been typically reported.^13,14,85,88,90^

Remarkably, thorough grinding of single crystals of **3** or vacuum evaporation of a dimethylformamide solution of **3** generates a material with much brighter luminescence. The predominantly amorphous nature of the solid is confirmed by powder XRD measurement (Figure S5). The crystal-to-amorphous phase transition and the decrease of the crystal size do not considerably affect the excitation spectra but red-shift the maxima of both emission bands for ca 20–25 nm, retaining their approximate ratio. This is accompanied by a nearly 7-fold increase in the total intensity (Φ_em_ = 0.06) and the disappearance of the fine structure of the dominating low-energy band. The latter indicates a more significant charge transfer character of the triplet state in the amorphous sample than that in bulk crystals. The gain in quantum yield arises from the suppression of nonradiative decay that is manifested by nearly an order of magnitude drop of the corresponding rate constant for amorphous **3** (Table 1). Despite the fact that the disruption of the lattice is expected to produce a less rigid local environment, the decrease in *k*_nr_ probably results from the disappearance of intermolecular interactions between organic chromophores (Figure 3), which could quench the luminescence in the neat crystal, making this effect opposite to the one termed “crystallization-induced emission”, e.g., in [PbX_2_(4,4′-bipyridine *N*-oxide)]*_n_* coordination polymers.^91^ The switch of weak fluorescence in crystals to stronger phosphorescence in the glass phase was observed for lead(II) alkanoates and explained by the shortening of intermolecular Pb–Pb distances.^92^ Importantly, the dominating phosphorescence of amorphous **3** indicates that it is an intrinsic molecular property but not an effect of crystal packing. Furthermore, it confirms an efficient assembly of this complex upon solvent removal, notwithstanding its complete dissociation in solution that opens a way for facile solution processing of these species. In terms of potential practical applications of this sort of compound, the improvement of the photophysical performance in an amorphous state could simplify the fabrication and handling of uniform films for optoelectronic devices.

The presence of a weak prompt fluorescence signal propounds that S_1_ → S_0_ radiative relaxation competes with relatively slow intersystem crossing, eventually induced by the heavy atom effect of the lead bromide fragment. The same phenomena were observed in the 77 K spectra for both amorphous **3** and crystalline **3** (Figure S6). Their emission intensities increase significantly by lowering the temperature, which suppresses the thermally induced nonradiative deactivations. For amorphous **3**, its low-temperature fluorescence and phosphorescence bands are blue-shifted ca 20 nm compared to those at room temperature and indicate excited-state destabilization at low temperatures, plausibly due to hindered intermolecular relaxation in the amorphous phase under cryogenic conditions. Support for this viewpoint is given by crystalline **3**, where the intermolecular relaxation is insignificant, and therefore, the fluorescence and phosphorescence peaks remain unchanged by varying the temperature.

**Figure 7:**
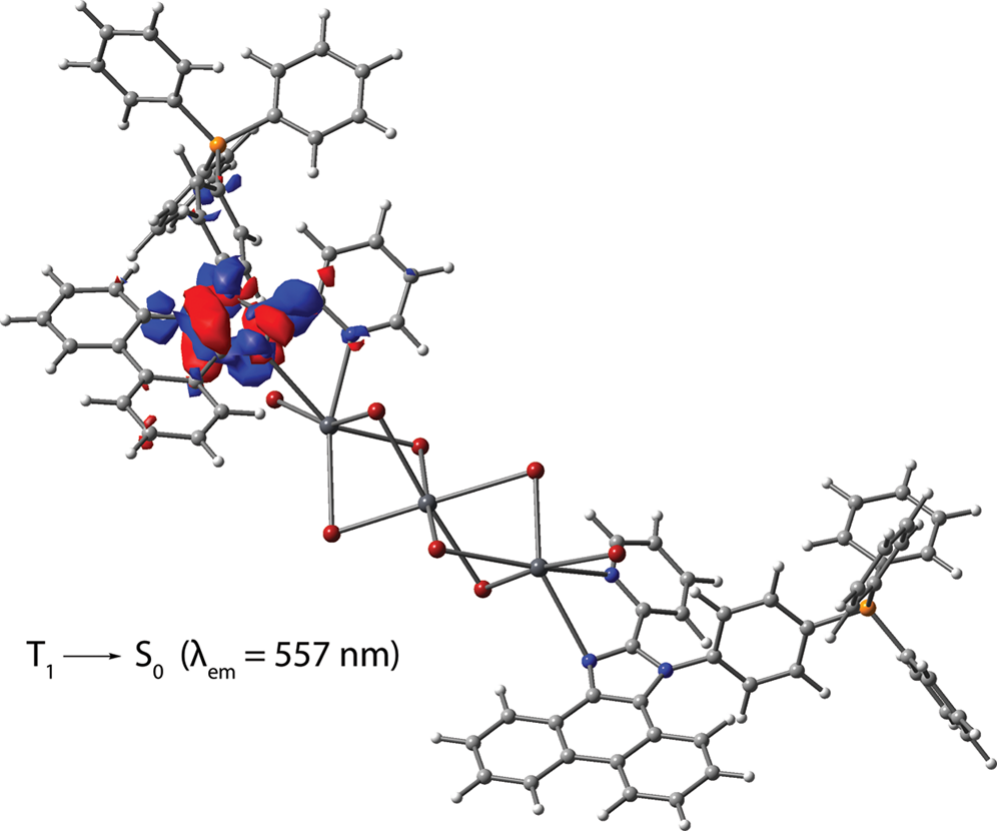
Electron density difference plot for the lowest energy triplet emission T_1_ → S_0_ in complex **3** (isovalue 0.002 a.u., DFT-LRC-ωPBEh method using the CPCM solvation model^75^ with toluene as a solvent, optimized T_1_ geometry). During the electronic transition, the electron density increases in the red areas and decreases in the blue areas.

Theoretical analysis of **3** was performed using the implicit CPCM solvation model^75^ in the DFT calculations because the geometry optimization did not converge in a vacuum environment. The inclusion of toluene as a solvent helped in reaching satisfactory optimization of the geometries of S_0_ and T_1_ states. As the use of solvent is not justified experimentally, because it is absent in the crystal structure of **3**, the character of the predicted S_0_ → S_1_ excitation yet remains ambiguous. Given the similarity of the experimental excitation spectra for compounds **1**–**3**, it is reasonable to assume photoinduced IL charge transfer transition (imidazole → phenylene) for **3** as well. The T_1_ state for **3** seems to be mostly insensitive to the choice of solvent, and the T_1_ → S_0_ emission is governed by the electronic transitions of one of the diimine ligands, which occur within the imidazole ring and the nearest bonds around it (Figure 7). The calculated wavelength for the phosphorescence of **3** (λ_calc_ = 557 nm, Table S3) matches well the observed ones. Regarding the intraligand triplet emission of **3**, it is worth mentioning that such behavior is rare among lead compounds. Long-lived organic phosphorescence, as a rule, with millisecond lifetimes, has been abundantly reported for some Pb(II) coordination polymers,^91^ constructed mainly from aromatic carboxylate blocks.^92−96^ Alternatively, efficient room-temperature phosphorescence of organic components has been realized in lead perovskites by introducing chromophore cations^29,97,98^ or sensitizing agents.^99^ However, on a molecular scale, complex **3** is one of the so far very few molecular lead compounds exhibiting phosphorescence at ambient temperature.^100,101^

## Conclusions

In this work, we have demonstrated the strategy of using the cationic diimine ligand, phosphonium-functionalized phenanthro-imidazolyl pyridine **LP**^**+**^Br, for the straightforward preparation of inorganic–organic hybrid complexes of zinc (**1**), cadmium (**2**), and lead (**3**). Compounds **1** and **2** represent zero-dimensional salts containing ligand-derived complex cations [**LP**^**+**^MBr_2_]^+^ and bromometalate anions [MBr_4_]^2–^. The lead bromide resulted in the assembly of a novel type of zwitterionic complex, in which the anionic cluster [Pb_3_Br_8_]^2–^ is stabilized by two coordinated motifs **LP**^**+**^. The analysis of the photophysical behavior of **1**–**3** in the solid state showed that the properties of **1** and **2** are governed by the intraligand charge transfer, resulting in moderate blue fluorescence, which is visibly enhanced versus the parent organic salt **LP**^**+**^Br. On the other hand, complex **3** demonstrates a weak fluorescence signal along with room-temperature phosphorescence, which is a very rare case for Pb(II) molecular compounds. The phosphorescence mainly originates from an intraligand transition, while the lead and bromine atoms act as external heavy elements to boost the spin–orbit coupling. Notably, weak luminescence of crystalline **3** is drastically enhanced in the amorphous state, retaining the dominating role of the triplet excited state. Essentially, it allows ascribing the observed photoemission of **3** to the molecular properties but not to the consequence of crystal packing. Thus, it potentially opens ways for new structural chemistry and for tuning the optical characteristics of related hybrid and zwitterionic species by employing positively charged organic ligands possessing different chromophore fragments and a variable number of cationic groups. Despite presenting a case study of one particular ligand, the described concept likely can be applied to a large selection of organic ligands that will allow generation of novel ionic crystalline materials, zwitterionic aggregates, and heterometallic systems with rich photophysical behavior. Although **3** undergoes complete dissociation in solution, it is efficiently assembled upon solvent removal, which is presumably facilitated by the electrostatic attraction of inorganic and organic blocks. No less important is the stability of **3** toward water that enables convenient fabrication, handling, and solution processing of the complex without special precautions.

## Supporting Information Available

Crystal data and structure refinement for **1**–**3;** selected structural parameters for **1**–**3**; and additional spectroscopic and computational data and figures (PDF)
