# Supplementary material for: Hybrid Inorganic–Organic Complexes of Zn, Cd, and Pb with a Cationic Phenanthro-diimine Ligand
Source: Inorg Chem. 2022 Nov 22;61(48):19220–31. doi: 10.1021/acs.inorgchem.2c02867 (PMC9727735; doi:10.1021/acs.inorgchem.2c02867)
Supplement: Supplementary file 1 — ic2c02867_si_001.pdf [file ic2c02867_si_001.pdf]

Supporting Information for

Hybrid inorganic-organic complexes of Zn, Cd and Pb with cationic phenanthro-diimine ligand

Diana Temerova,[a] Tai-Che Chou,[b] Kristina S. Kisel,[a] Toni Eskelinen,[c] Niko Kinnunen,[a] Janne Jänis,[a] Antti J. Karttunen,[c] Pi-Tai Chou,[b]* Igor O. Koshevoy[a]**

[a] Department of Chemistry, University of Eastern Finland, Joensuu, 80101, Finland

[b] Department of Chemistry, National Taiwan University, Taipei 106, Taiwan

[c] Departmentof Chemistry and Materials Science, Aalto University, Aalto, 00076, Finland

E-mail: antti.karttunen@aalto.fi; chop@ntu.edu.tw; igor.koshevoy@uef.fi;

Table S1. Crystal data and structure refinement for **1**–**3**. S2

Table S2. Selected bond length and angles for **1**–**3**. S3

Figure S1. ^{1}H NMR spectra of ligand **LP**$^{+}$Br and complexes **1**, **2**. S4

Figure S2. A: UV-vis absorption and emission spectra of **LP**$^{+}$Br (298 K). S4

Table S3. Calculated electronic absorption and emission data for ligand **LP**$^{+}$Br and compounds **1**–**3**. S5

Figure S3. Electron density difference plots for the lowest energy excitation $S_0 \rightarrow S_1$ and emission $S_1 \rightarrow S_0$ in the cation **LP**$^{+}$. S5

Figure S4. Electron density difference plots for the lowest energy excitations $S_0 \rightarrow S_1$ emission $S_1 \rightarrow S_0$ in the dication [**LP**$^{+}$CdBr$_2$]$_2^{2+}$. S6

Figure S5. Powder XRD patterns for complex **3**. S7

Figure S6. Solid state emission spectra for complex **3** at 77 K. S8

Table S1. Crystal data and structure refinement for **1**–**3**.

**Identification code**	**1**	**2**	**3**
CCDC	2195809	2195808	2195810
Empirical formula	$C_{178}H_{127}Br_{16}N_{13}P_4Zn_6$	$C_{90}H_{65}Br_8Cd_3N_7P_2$	$C_{88}H_{62}Br_8N_6P_2Pb_3$
Formula weight	4242.58	2282.91	2526.22
Temperature (K)		150(2)	
Wavelength (Å)		0.71073	
Crystal system	Triclinic	Triclinic	Monoclinic
Space group	$P\bar{1}$	$P\bar{1}$	*C*2/*c*
Unit cell dimensions			
a (Å)	13.6686(10)	9.5730(13)	32.5145(15)
b (Å)	17.4156(13)	21.412(3)	15.7172(7)
c (Å)	18.0398(13)	24.749(3)	16.9814(8)
a (°)	77.487(2)	111.165(3)	90
β (°)	83.607(2)	99.442(3)	107.3770(10)
γ (°)	82.321(2)	97.972(3)	90
Volume ($Å^3$)	4139.6(5)	4557.4(10)	8282.1(7)
Z	1	2	4
ρ_{calc} (Mg/m^3)	1.702	1.664	2.026
μ (mm^{-1})	4.812	4.282	10.031
F(000)	2086	2216	4752
Crystal size (mm^3)	0.338 × 0.245 × 0.224	0.959 × 0.381 × 0.074	0.302 × 0.224 × 0.110
θ range for data collection (°)	1.509 to 28.978	1.701 to 28.000	2.379 to 31.030
Index ranges	-17<=h<=18, -22<=k<=23, -24<=l<=22	-12<=h<=11, -28<=k<=28, -32<=l<=32	-46<=h<=47, -22<=k<=22, -24<=l<=18
Reflections collected	62065	74998	40033
Independent reflections	20972 [R(int) = 0.0338]	21515 [R(int) = 0.0423]	12459 [R(int) = 0.0318]
Completeness to θ = 25.242°	99.4 %	99.2 %	99.8 %
Absorption correction	Numerical	Numerical	Numerical
Max. and min. transmission	0.412 and 0.293	0.742 and 0.105	0.405 and 0.152
Refinement method		Full-matrix least-squares on F^2	
Data/ restrains/ parameters	20972 / 6 / 992	21515 / 54 / 1014	12459 / 0 / 483
GOOF on F^2	1.074	1.163	1.025
Final R indices [I>2σ(I)][a]	R1 = 0.0621, wR2 = 0.1819	R1 = 0.1112, wR2 = 0.2718	R1 = 0.0310, wR2 = 0.0571
R indices (all data)	R1 = 0.0892, wR2 = 0.1952	R1 = 0.1242, wR2 = 0.2778	R1 = 0.0517, wR2 = 0.0617

Largest diff. peak and hole (e.Å^{-3})	2.091 and -1.648	2.509 and -1.679	1.512 and -2.390

[a] $R_1 = \Sigma||F_o| - |F_c||/\Sigma|F_o|$; $wR2 = [\Sigma [w(F_o^2 - Fc^2)^2]/ \Sigma[w(F_o^2)^2]]^{1/2}$

Table S2. Selected bond length and angles for **1–3**.

**1**		**2**		**3**	
**Bond lengths, Å**					
N(1)-Zn(1)	2.055(5)	N(1)-Cd(1)	2.292(11)	N(1)-Pb(1)	2.642(3)
N(2)-Zn(1)	2.051(5)	N(2)-Cd(1)	2.385(11)	N(2)-Pb(1)	2.759(3)
N(4)-Zn(2)	2.063(5)	Br(1)-Cd(1)	2.528(2)	Br(1)-Pb(1)	2.8991(3)
N(5)-Zn(2)	2.056(5)	Br(2)-Cd(2)	2.541(2)	Br(2)-Pb(1)	2.8049(4)
Zn(1)-Br(1)	2.3381(11)	Br(3)-Cd(1)	2.6403(19)	Br(3)-Pb(2)	2.9932(4)
Zn(1)-Br(2)	2.3616(10)	Br(3)-Cd(2)	2.8605(17)	Br(3)-Pb(1)	3.0893(4)
Zn(2)-Br(4)	2.3316(10)	Br(4)-Cd(2)	2.6137(18)	Br(4)-Pb(2)	2.7250(5)
Zn(2)-Br(3)	2.3594(10)	Br(4)-Cd(1)	2.8024(17)	Br(4)-Pb(1)	3.2608(8)
Zn(3)-Br(5)	2.4129(10)	Br(5)-Cd(3)	2.5884(19	Pb(1)-Pb(2)	4.1680(7)
Zn(3)-Br(7)	2.4148(10)	Br(6)-Cd(3)	2.5817(18)	-	-
Zn(3)-Br(8)	2.4221(12)	Br(7)-Cd(3)	2.6211(18)	-	-
Zn(3)-Br(6)	2.4296(11)	Br(8)-Cd(3)	2.5742(18)	-	-
**Bond angles, °**					
N(2)-Zn(1)-N(1)	80.5(2)	N(1)-Cd(1)-N(2)	72.2(4)	N(1)-Pb(1)-N(2)	62.61(8)
N(5)-Zn(2)-N(4)	80.6(2)	Br(1)-Cd(1)-Br(3)	120.37(8)	Br(2)-Pb(1)-Br(1)	103.689(12)
Br(1)-Zn(1)-Br(2)	113.97(4)	Br(1)-Cd(1)-Br(4)	93.17(6)	Br(2)-Pb(1)-Br(3)	90.851(12)
Br(4)-Zn(2)-Br(3)	111.80(4)	Br(3)-Cd(1)-Br(4)	86.86(5)	Br(1)-Pb(1)-Br(3)	164.329(12)

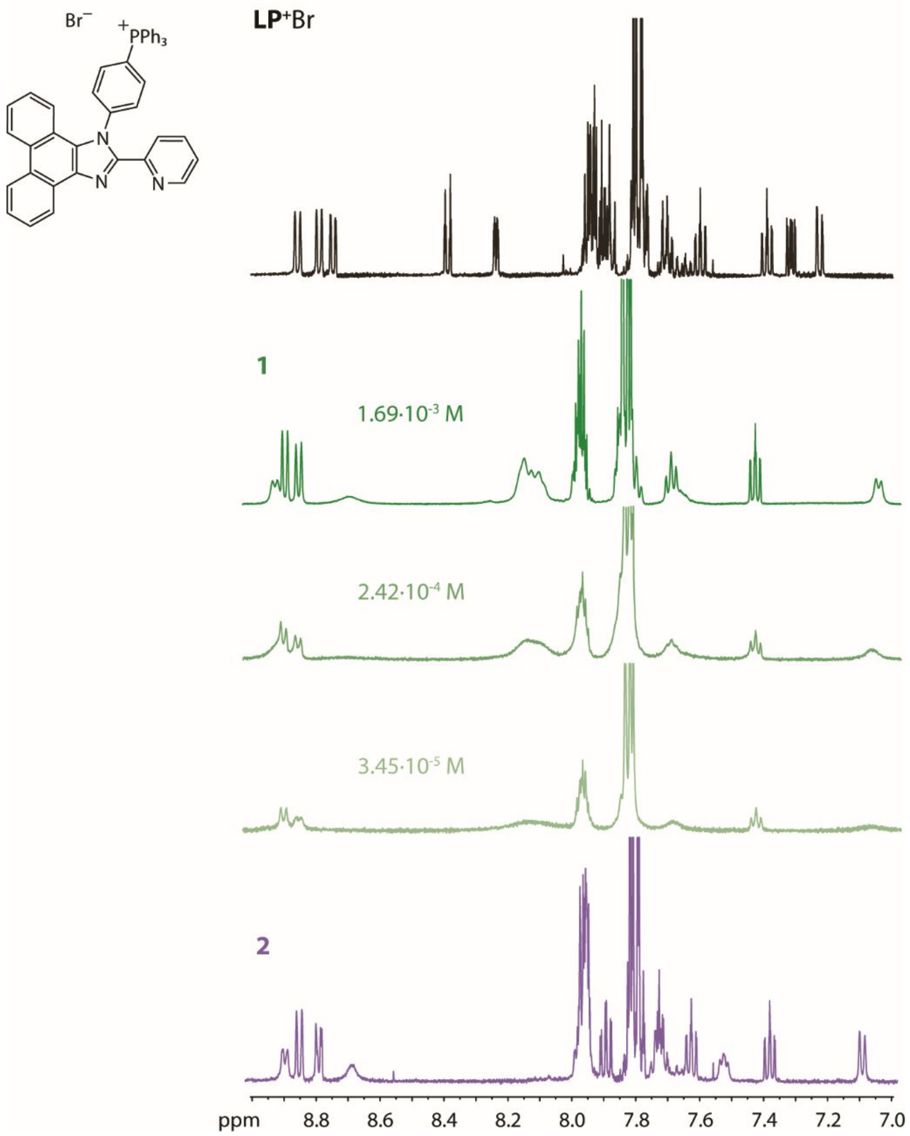

Figure S1. ^{1}H NMR spectra of ligand **LP**+Br (black) and of complexes **1** (green, at different concentrations) and **2** (acetonitrile-d_3, 298 K).

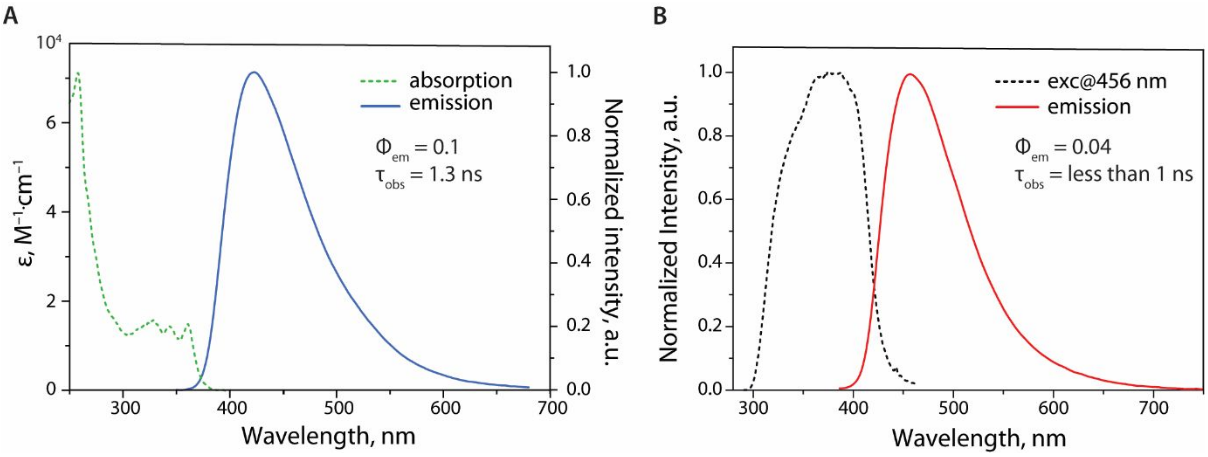

Figure S2. A: UV-vis absorption and emission (λ_{exc} = 300 nm) spectra of **LP**$^+$Br (dichloromethane, 298 K); B: normalized excitation and emission (λ_{exc} = 355 nm) spectra of **LP**$^+$Br in the solid state (298 K).

Table S3. Calculated electronic absorption and emission data for ligand **LP**$^+$Br and compounds **1**–**3**.

Cation	$S_0 \rightarrow S_1$ excitation		$S_1 \rightarrow S_0$ emission	
	E, eV	λ, nm	*E*, eV	λ, nm
**LP**$^+$	3.65	340	2.93	424
[**LP**$^+$ZnBr$_2$]$^+$ (**1**)	3.50	354	2.78	446
[**LP**$^+$CdBr$_2$]$^+$ (**2**) (half model)[a]	3.67	338	2,83	438
			$T_1 \rightarrow S_0$ emission	
**3** (in toluene)	3.92	317	2.23	557

[a] Half model for the cation of **2** was satisfactorily used for the sake of simplicity because the $S_0 \rightarrow S_1$ excitation in the full model [**LP**$^+$CdBr$_2$]$_2^{2+}$ involves contribution from half of it (see Figure S4).

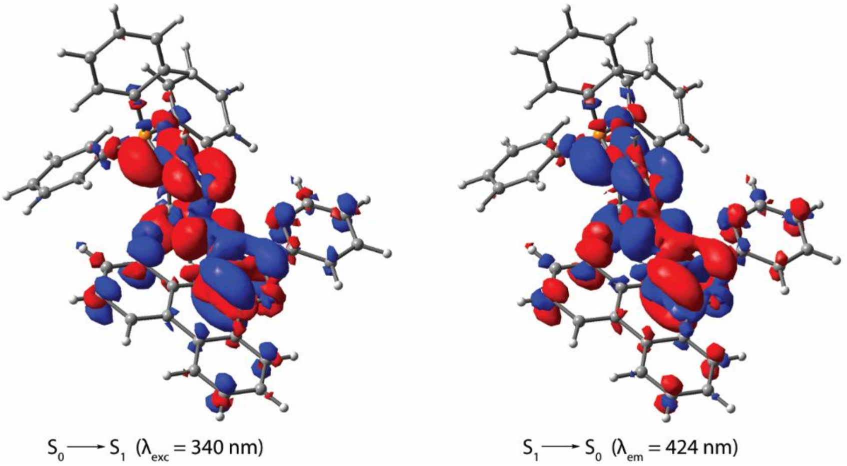

Figure S3. Electron density difference plots for the lowest energy excitation $S_0 \rightarrow S_1$ and emission $S_1 \rightarrow S_0$ in the cation **LP**$^+$ (isovalue 0.002 a.u., DFT-LRC-ωPBEh method, optimized S_0 and S_1 geometries). During the electronic transition, the electron density increases in the red areas and decreases in the blue areas.

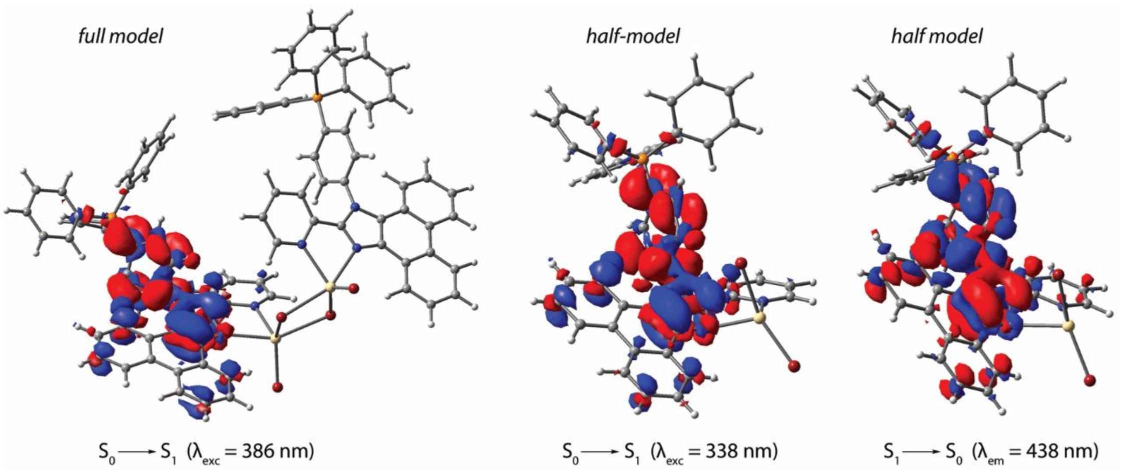

Figure S4. Electron density difference plots for the lowest energy excitations $S_0 \rightarrow S_1$ in the dication [**LP**$^+$CdBr$_2$]$_2^{2+}$ and its half model [**LP**$^+$CdBr$_2$]$^+$, and for the emission $S_1 \rightarrow S_0$ for half model cation [**LP**$^+$CdBr$_2$]$^+$ (isovalue 0.002 a.u., DFT-LRC-ωPBEh method, optimized S_0 and S_1 geometries). During the electronic transition, the electron density increases in the red areas and decreases in the blue areas.

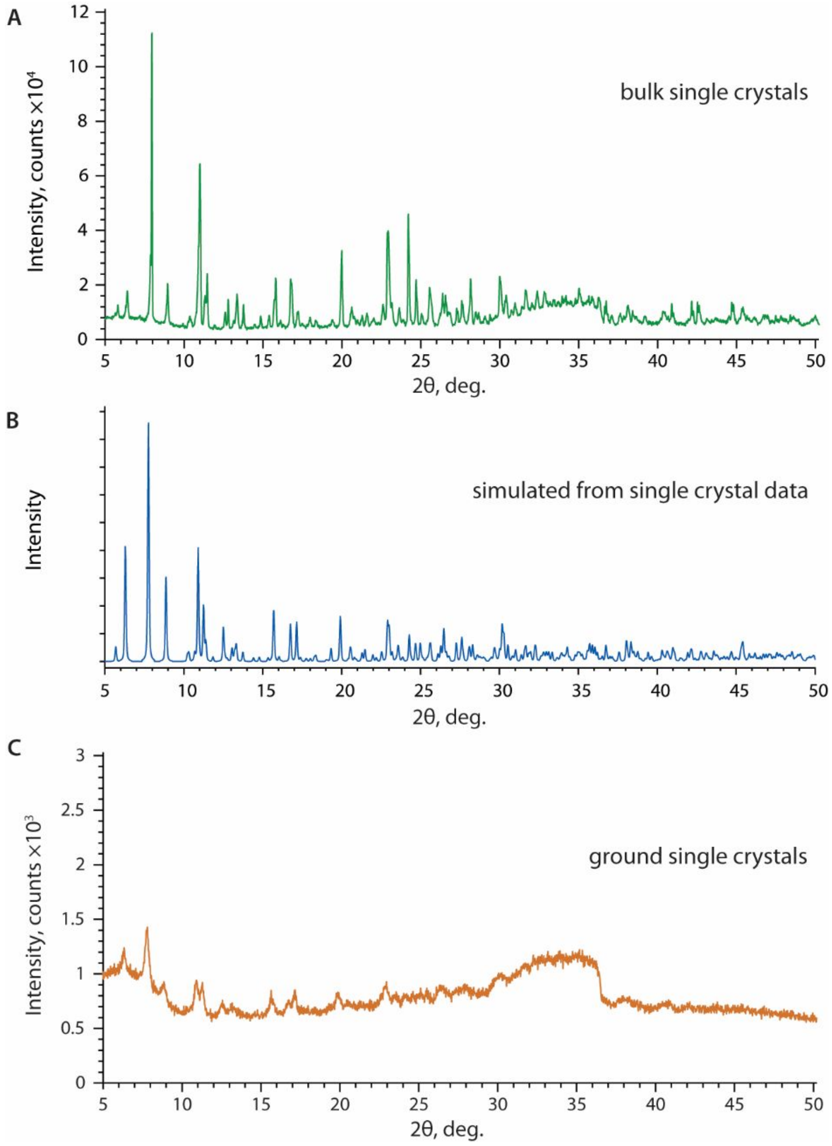

Figure S5. Powder XRD patterns for complex **3**: (A) bulk single crystals, (B) simulated pattern from single crystal data, (C) ground single crystals (the region of 2θ between 28–38° shows an increased background, at high voltages the energy resolution of the scintillation counter is insufficient to discriminate this).

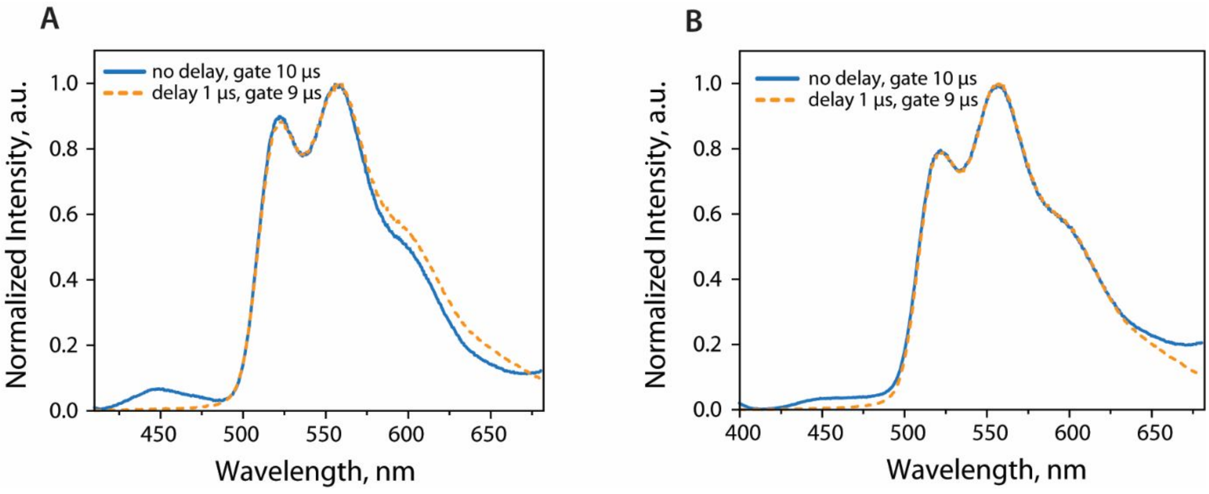

Figure S6. Solid state emission spectra for complex **3** at 77 K. The broad band at 450 nm and the vibronic progression at 520, 560, 610 nm are assigned as fluorescence and phosphorescence, respectively. (A) microcrystalline form $\mathbf{3}_{mc}$; (B) single crystals $\mathbf{3}_{sc}$. Blue line: spectra captured right after the laser pulse with 10 µs gating time; orange dashed line: spectra captured 1 µs after the laser pulse with 9 µs gating time.
